# Vancomycin-Induced Leukocytoclastic Vasculitis: A Rare Complication From a Commonly Used Medication

**DOI:** 10.7759/cureus.36532

**Published:** 2023-03-22

**Authors:** Inderpal Singh, Harish Gidda, Bola Nashed

**Affiliations:** 1 Internal Medicine, Ascension St. John Hospital, Detroit, USA

**Keywords:** antibiotic reaction, leukocytoclastic vasculitis (lcv), palpable purpura, medication induced vasculitis, small vessel vasculitis

## Abstract

Leukocytoclastic vasculitis (LCV) is a cutaneous small vessel vasculitis that is characterized by the development of a non-blanching palpable purpura. Diagnosis is made by skin biopsy and histopathology which shows subepidermal acantholysis with dense neutrophilic infiltrate leading to fibrinoid necrosis of the dermal blood vessels. Etiology is generally idiopathic in most cases but secondary causes include chronic infections, malignancies, systemic autoimmune conditions, and medication use. Treatment involves supportive measures in the case of idiopathic LCV, and treatment of the offending condition or agent in LCV due to a secondary cause.

A 59-year-old male presented with purulent ulcers on the plantar surface of the right foot. Radiograph of the right foot showed soft tissue swelling without evidence of osteomyelitis. Empiric antibiotic treatment with vancomycin was initiated. A wound culture was obtained from the purulent drainage which grew positive for methicillin-resistant *Staphylococcus aureus* (MRSA). On the fourth day of treatment with vancomycin, multiple symmetric, purpuric lesions arose on the patient's trunk and extremities. Skin biopsy with histopathology showed subepidermal acantholysis with neutrophil-predominant inflammatory infiltrate consistent with leukocytoclastic vasculitis. Vancomycin was discontinued and the patient's exanthem began to regress, with full resolution after 30 days post withdrawal of the antibiotic.

## Introduction

Vancomycin is a widely used antibiotic for the treatment of infections caused by methicillin-resistant *Staphylococcus aureus* (MRSA). Vancomycin has been commonly associated with cutaneous and hypersensitivity drug reactions such as “red-man syndrome”, linear IgA bullous dermatosis, drug rash eosinophilia and systemic symptoms (DRESS) syndrome, along with Stevens-Johnson/toxic epidermal necrolysis [1]. In spite of this, vancomycin-induced vasculitides have rarely been reported in the literature. Here, we highlight a unique presentation of vancomycin-induced leukocytoclastic vasculitis in a patient with purulent cellulitis. This article was previously presented as a meeting abstract at the American College of Physicians National Internal Medicine Meeting on March 23-25, 2020.

## Case presentation

A 59-year-old male initially presented with pain and a non-healing ulcer on the plantar surface of the right foot. The patient's past medical history is significant for hypertension, hyperlipidemia, and type II diabetes mellitus with complications of peripheral neuropathy. Initial vital signs were significant for sinus tachycardia with a rate of 103 beats per minute. On physical exam, the patient's right foot was swollen, erythematous and warm to the touch. A 2 cm diameter ulcer was noted on the plantar surface of the right foot with surrounding erythema and purulent drainage. Initial laboratory work up was obtained and outlined in Table 1.

**Table 1:** Initial laboratory workup

Test	Results	Reference Range
White Blood Cell Count	13.78	4.00-11.00 x 10^3^/uL
Hemoglobin	13.2	12.0-16.0 gm/dL
Platelet Count	403	150-400 K/mcL
Eosinophil Count	5.4	0-4%
Creatinine	1.03	0.5-1.1 mg/dL
Blood Urea Nitrogen	25	6-23 mg/dL

**Figure 1:**
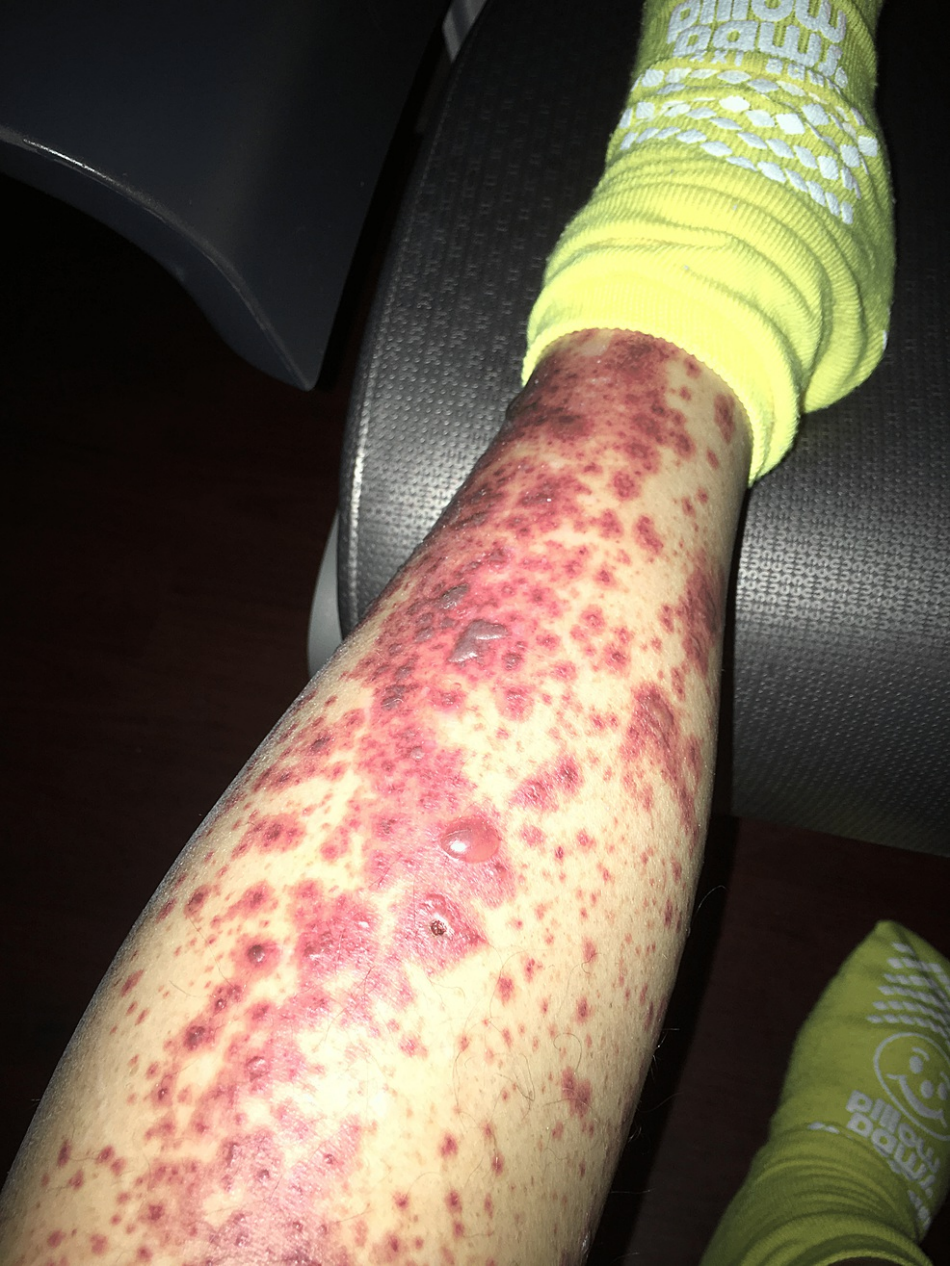
The erythematous palpable purpura that began four days post initiation of vancomycin as seen on the left lower extremity. This exanthem was also noted on the trunk as well as the right lower extremity.

Imaging with a radiograph of the right foot was done which showed soft tissue swelling in the plantar surface consistent with cellulitis without periosteal reaction and cortical irregularity of the bone. Due to the patient experiencing purulent drainage from the ulcer on the right foot, the patient was empirically started on antibiotic treatment with intravenous vancomycin. Blood and wound cultures were obtained at the time empiric antibiotics were initiated. Blood cultures showed no growth after 24-48 hours, while wound cultures grew positive for MRSA after roughly 24-36 hours. The patient was tolerating treatment well and the clinical picture was improving, the patient's pain in the right foot also improved as well through the clinical course. On the fourth day of therapy with intravenous vancomycin, the patient developed a symmetrically diffuse, erythematous, purpuric rash on the trunk, along with on the bilateral upper and lower extremities (Figure 1).

Further workup to elucidate the possible systemic etiology of the rash was done and summarized in Table 2.

**Table 2:** Workup to elucidate the possible systemic cause of palpable purpura. HIV - Human immunodeficiency virus, Ab - Antibody, DNA - Deoxyribonucleic acid, PR3 – Proteinase 3, ANCA – Antineutrophil cytoplasmic antibodies, MPO – Myeloperoxidase

Test	Results	Reference Range
C3 Complement	188	79-152 mg/dL
C4 Complement	53	16-38 mg/dL
Hepatitis A, B core and C total antibody	Negative	Negative
HIV-1, HIV-2 Ab	Non Reactive	Non Reactive
Antinuclear antibody	Negative	Negative
Double stranded DNA Ab	Negative	Negative
PR3-ANCA	Not detected	Not detected
MPO-ANCA	Not detected	Not detected

**Figure 2:**
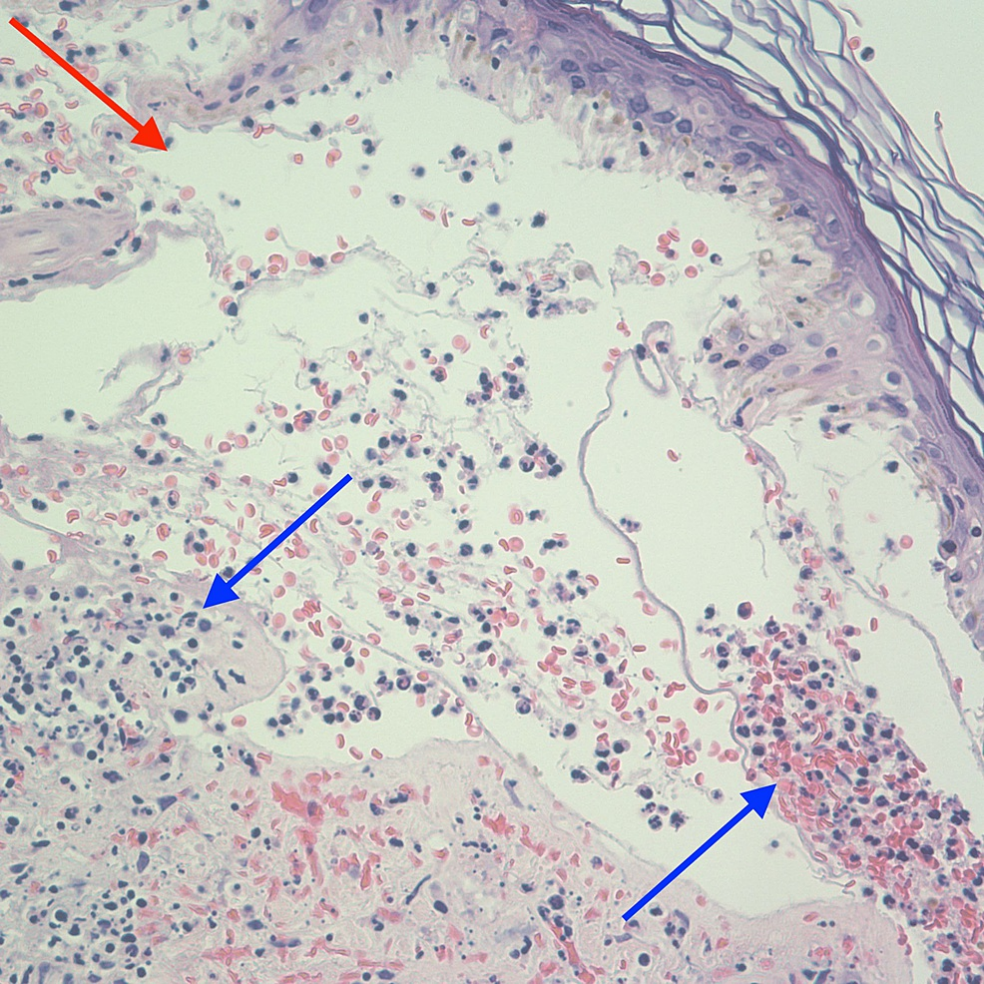
Hematoxylin and eosin stained image at 100x magnification from biopsy of the exanthem site showing subepidermal acantholysis (red arrow) with predominantly neutrophil inflammatory cell infiltrate (blue arrow).

**Figure 3:**
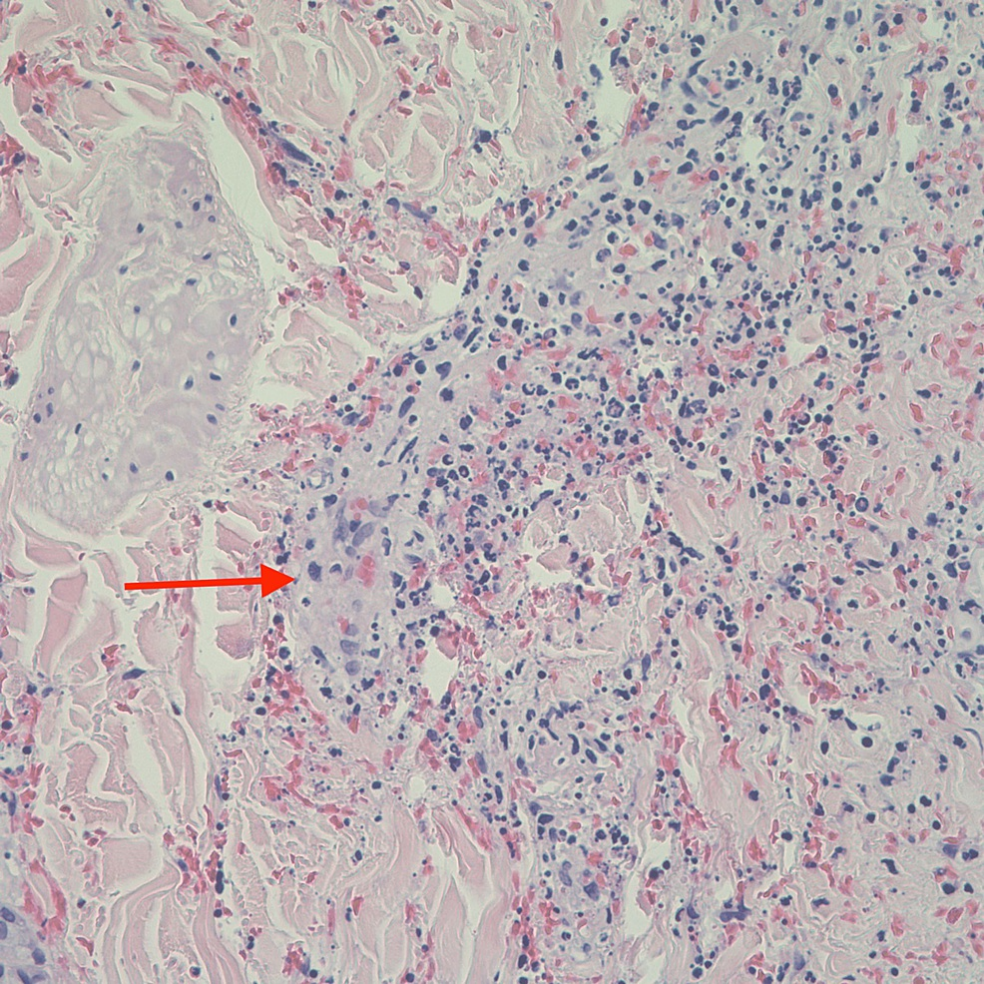
Hematoxylin and eosin stained image at 100x magnification from biopsy of the exanthem site showing dense neutrophilic infiltrate and subsequent fibrinoid necrosis (red arrow).

With the workup for systemic causes being negative, a skin biopsy from the exanthem site was done and showed subepidermal blister formation with dense neutrophilic infiltrate (Figure 2) and fibrinoid necrosis of the dermal blood vessels, findings which are consistent with leukocytoclastic vasculitis (LCV) (Figure 3).

**Figure 4:**
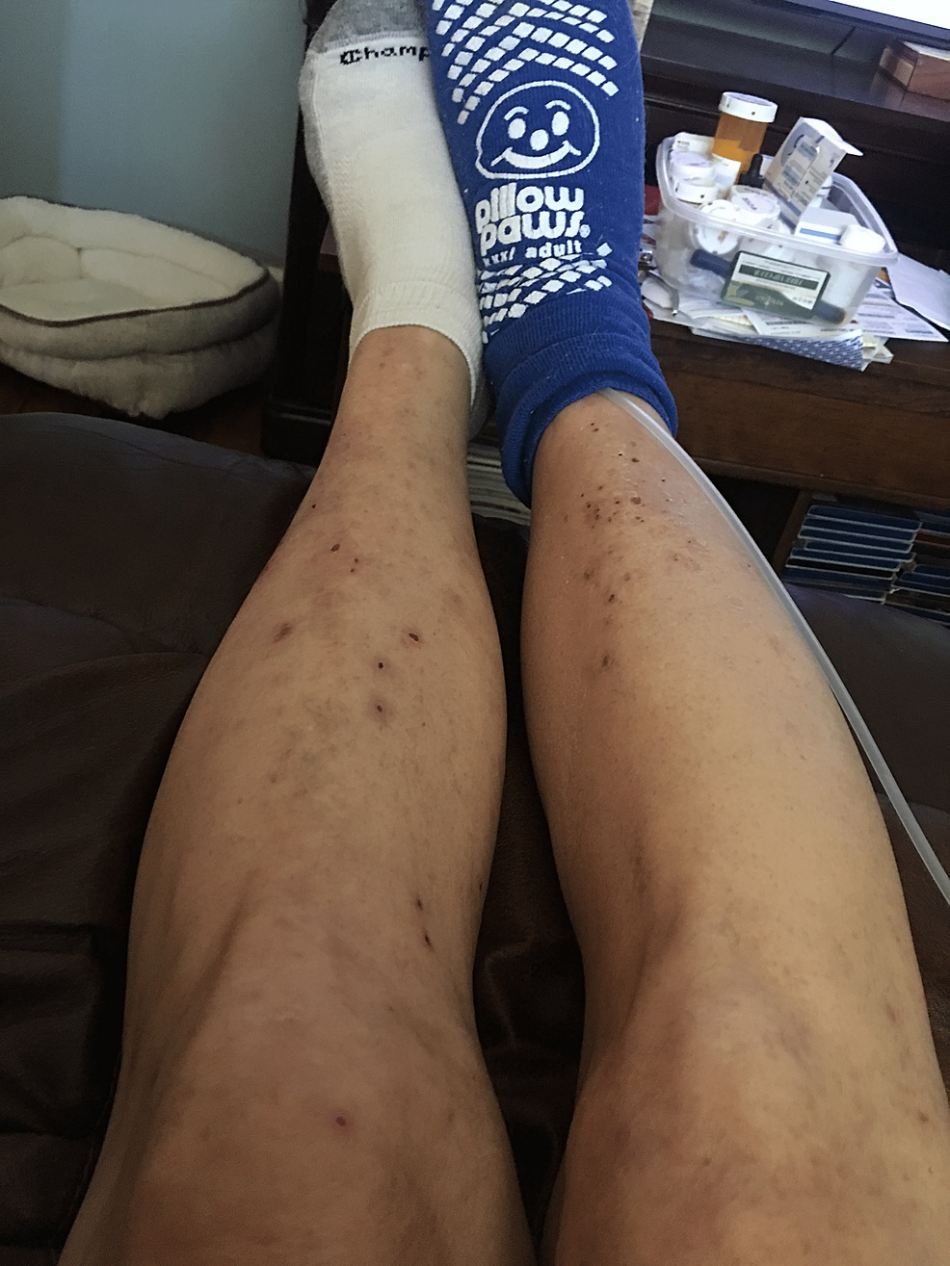
Regression and resolution of the exanthem three to four weeks after withdrawal of vancomycin.

No systemic involvement was appreciated as the patient's platelet count and creatinine levels were measured to be 403 K/mcL and 1.03 mg/dL respectively. Upon undergoing a medical reconciliation for the patient, intravenous vancomycin was discontinued and the antibiotic regimen was continued with oral doxycycline for the remainder of the treatment course. Upon discontinuation of vancomycin, the patient's rash began to regress (Figure 4). The patient was discharged with oral antibiotics to complete the full antibiotic course. Upon contacting the patient post discharge, the exanthem had fully resolved roughly 30 days after vancomycin had been discontinued.

## Discussion

LCV is a histopathological term that generally describes a small vessel vasculitis that has predominantly cutaneous involvement. The most common clinical finding of LCV is the presence of a cutaneous non-blanching palpable purpura, although exclusive erythema, maculopapular lesions, or petechiae have also been noted in the literature [2-3]. Histological analysis of the cutaneous lesions in LCV typically shows sub-epidermal acantholysis with a dense inflammatory infiltrate predominantly composed of neutrophils, leading to a subsequent release of lysosomal enzymes causing a fibrinoid necrosis of the vasculature [4-5].

LCV is commonly idiopathic in nature, although secondary causes of LCV that have been seen in the literature include acute infections, systemic autoimmune conditions, malignancies, and medication use. Several medications have been known to cause LCV including but not limited to antibiotics such as vancomycin and beta-lactams, diuretics, non-steroidal anti-inflammatory drugs (NSAIDs), beta-blockers, and anti-epileptic medications among many others. Through medication-induced LCV, the onset of cutaneous symptoms typically occurs within 24 hours to one month post administration of the medication [5]. It was presumed that vancomycin was the cause and etiology for the exanthem as workup with negative blood cultures ruled out systemic infection. Furthermore, the patient's history, initial presentation, and clinical course did not indicate a malignant etiology for the patient's symptoms. The timing of the development of the exanthem, which appeared in our patient on day four after initiation of vancomycin, along with the regression and resolution once vancomycin was discontinued, made antibiotic-induced LCV the most probable cause.

Treatment of LCV is determined by the underlying etiology. Most idiopathic cases of LCV are treated with supportive measures such as compression stockings, leg elevation, and antihistamines. Medications such as corticosteroids and immunosuppressants such as methotrexate, azathioprine, and mycophenolate mofetil can also be used for cases in which supportive measures fail [6]. For LCV secondary to a known cause such as a connective tissue disorder, malignancy, chronic infection, or medication use, treatment or cessation of the offending condition or agent is crucial for the resolution of symptoms [5]. This was seen in our patient as well, as withdrawal of vancomycin and initiation of doxycycline for the remainder of the antibiotic regimen led to a regression of the patient's symptoms, with complete resolution roughly 30 days after discharge from the hospital.

## Conclusions

LCV is a cutaneous small vessel vasculitis that predominantly presents with a non-blanching palpable purpura. Majority of cases of LCV are idiopathic, with secondary causes being due to chronic infections, systemic autoimmune conditions, malignancies, and medication use such as antibiotics. Although rare, this case highlights that clinicians should carefully observe patients being treated with vancomycin. Due to increasing resistance rates to antibiotics, the use of broad-spectrum antibiotics such as vancomycin is expected to increase. As a result, clinicians should be aware of vancomycin as a potential cause of this complication.
